# Relative effectiveness in genetic gain from genomic selection of candidate dams versus genomic selection of their progeny

**DOI:** 10.3168/jdsc.2024-0705

**Published:** 2025-05-01

**Authors:** D.P. Berry, T.B. Murphy

**Affiliations:** 1 Teagasc, Animal & Grassland Research and Innovation Centre, Moorepark, Fermoy, Co. Cork, P61 P302, Ireland; 2 School of Mathematics and Statistics, University College Dublin, Dublin, D04 V1W8, Ireland

## Abstract

The ability to genomically predict the genetic merit of individuals coupled with the widespread availability of sex-sorted semen is causing a paradigm shift in global animal breeding. Many dairy producers wish to minimize the number of surplus dairy-bred calves born and, in turn, maximize the proportion of beef-on-dairy matings. The aim could be to select the number of candidate dams to be just sufficient to generate ample dairy-bred replacement progeny; using this strategy, minimal scope exists for selection within the resulting progeny cohort. However, the strategy of applying most of the selection pressure on the dam (and sire) ignores the contribution of the random segregation of genetic material during gametogenesis. The hypothesis of this study was that too strict selection within the candidate dam population for mating to dairy-sire semen with minimal opportunity for female progeny selection would affect genetic gain relative to generating surplus female progeny from which selection could then be exercised. Deterministic approaches were developed, complemented with simulations, where different intensity of selection in the dam and female progeny cohorts were imposed. Relative to the selection of the top ranking 10%, 20%, 30%, 40%, and 50% of candidate dams based on their genomic evaluation with 60% reliability, no preselection of dams coupled with genomically selecting the top ranking 10% to 50% of the female progeny born (also reliability of 60%) resulted in female progeny that were expected to, on average, have a true breeding value 0.58, 0.46, 0.38, 0.32, and 0.26 genetic SD higher. Benefits also existed from combining genomic selection within the cohort of candidate dams followed by genomic selection within the resulting female progeny. In most cases, herds will seek to generate ∼30% of their herd size as replacement heifers; based on the population parameters used, in such a scenario there is a clear benefit from selecting the top 60% of the candidate dams with the further gains thereafter being relatively small. This study provides the necessary equations to help producers make herd breeding decisions; the actual relative difference among different scenarios depends on the used population parameters.

## Highlights

• Dairy producers are minimizing the number of dams used to generate replacements.
• Selection of just parents does not maximize the progeny's genetic merit.
• Selection of female progeny is superior to selection of just candidate dams.
• Many factors dictate the optimal trade-off between dam and heifer selection.

## Graphical Abstract

**Figure:**
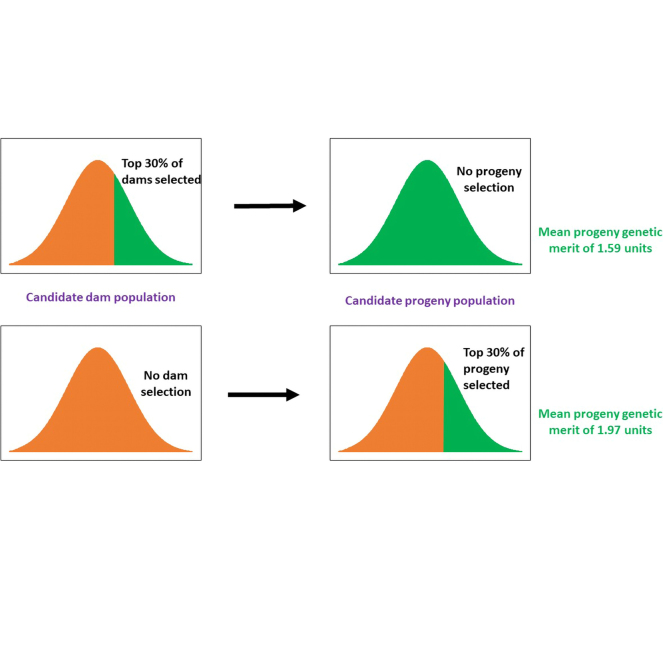
**Summary:** Technological advancements in genotyping and semen sex-sorting processes have contributed to a reevaluation of herd breeding programs to generate sufficient females to sustain the mature herd. There is a trend in many dairy herds to minimize the number of candidate dams to generate dairy female replacements. This strategy ignores the contribution of the random segregation of genetic material during gametogenesis. The results from deterministic equations and simulations used in this study highlight the benefit in genetic gain of generating female progeny surplus to requirement and then selecting from within this cohort.

The rate of genetic gain in a (sub)population is a function of, among other factors, the intensity of selection and the accuracy of this selection (Rendel and Robertson, 1950). Genomic evaluations, which are now commonplace in many developed breeding programs, use genome-wide markers to predict the true genetic merit of individuals (VanRaden, 2008); the effect is greater accuracy of selection at a younger age. Delivering this greater accuracy is a direct consequence of being able to estimate the Mendelian sampling term made possible through genotyping. Concurrent advancements in the sex sorting of semen is contributing to a greater uptake of this technology globally, especially in dairy cow populations (Seidel and DeJarnette, 2022). All else being equal, and assuming minimal impact of sexed-semen on conception rate as can exist in some herds (Drake et al., 2020), the need to serve dams with dairy-sire semen to produce a sufficient number of replacement cows is reduced. This is leading to recommendations to select fewer candidate dams of the next generation of dairy replacements.

During gametogenesis, germ cells undergo meiosis to produce haploid cells that, in turn, develop into gametes. During the process of meiotic division, each pair of chromosomes can exchange genetic material and a random selection of the parental chromosomes are chosen for each gamete. By solely selecting the candidate dams, the segregation randomness associated with gametogenesis cannot be fully captured. The hypothesis of this study, therefore, was that choosing a candidate dam population larger than required to generate replacements, coupled with genomic selection of the subsequent female progeny, would deliver faster genetic gain in a herd than selection solely of the candidate dam population sufficient in size to generate the required number of female progeny. A deterministic approach was developed to be sufficiently generic to most populations where parameters such as the accuracy of genomic evaluations, sire selection intensity, pregnancy rate, litter size, required number of replacements, and progeny mortality rate could be altered. A simulation was also run based on a case study representing a typical genotyped dairy herd adopting a sexed-semen dairy-sire breeding program.

To simplify the simulations, it was assumed that every insemination with sexed semen resulted in pregnancy, and that every female calf born will eventually reproduce herself without any losses or failures in the process. The sexed semen was assumed to only produce female offspring. Each of these assumptions could later easily be altered and integrated into the presented calculations. For the purpose of the present study, the trait or index in the dam population was set to have an average true breeding value (**TBV**) of 0 with a SD of 1. The candidate sires were selected from a standardized normal distribution but only from the top end of this distribution to create an EBV variance of 0.05.

The expected mean (μ) and (co)variance (σ^2^) of the TBV and (genomic) EBV can be represented as

[1] $$\left(\begin{array}{c} \mathrm{TBV}\\ \mathrm{EBV} \end{array}\right)\;∼N\left(\left(\begin{array}{c} \mu_{\mathrm{TBV}}\\ \mu_{\mathrm{EBV}} \end{array}\right),\left(\begin{array}{cc} \sigma_{\mathrm{TBV}}^{2} & \sqrt{\mathrm{Rel}}\sigma_{\mathrm{TBV}}\sigma_{\mathrm{EBV}}\\ \sqrt{\mathrm{Rel}}\sigma_{\mathrm{TBV}}\sigma_{\mathrm{EBV}} & \sigma_{\mathrm{EBV}}^{2} \end{array}\right)\right),$$

where Rel is the reliability of the (genomic) evaluation. The expected mean and variance of the TBV of a population conditional on its respective EBV can be expressed as

[2] $$\begin{array}{c} \left(\mathrm{TBV}|\mathrm{EBV}\right)=\mu_{\mathrm{TBV}}+\;\frac{\sqrt{\mathrm{Rel}}\sigma_{\mathrm{TBV}}\sigma_{\mathrm{EBV}}}{\sigma_{\mathrm{EBV}}^{2}}\left(\mathrm{EBV}-\mu_{\mathrm{EBV}}\right)\;\\ =\mu_{\mathrm{TBV}}+\;\frac{\sqrt{\mathrm{Rel}}\sigma_{\mathrm{TBV}}}{\sigma_{\mathrm{EBV}}}\left(\mathrm{EBV}-\mu_{\mathrm{EBV}}\right), \end{array}$$

[3] $$\left(\mathrm{TBV}|\mathrm{EBV}\right)=\;\sigma_{\mathrm{TBV}}^{2}-\;\frac{\mathrm{Rel}\sigma_{\mathrm{TBV}}^{2}\sigma_{\mathrm{EBV}}^{2}}{\sigma_{\mathrm{EBV}}^{2}}=\;\left(1-\mathrm{Rel}\right)\sigma_{\mathrm{TBV}}^{2}.$$

The expected mean and variance of the EBV of a population which has been subjected to selection on a minimum threshold EBV (i.e., truncated at point a on the distribution of EBV) can be defined as

[4] $$\left(\mathrm{EBV}|\mathrm{EBV}>a\right)=\mu_{\mathrm{EBV}}+\;\frac{\phi (\alpha )}{1-\Phi (\alpha )}\sigma_{\mathrm{EBV}},$$

[5] $$\left(\mathrm{EBV}|\mathrm{EBV}>a\right)=\;\sigma_{\mathrm{EBV}}^{2}\left(1+\frac{\alpha \phi (\alpha )}{1-\Phi (\alpha )}-{\left(\frac{\phi (\alpha )}{1-\Phi (\alpha )}\right)}^{2}\right),$$

where $\alpha =\frac{a-\mu_{\mathrm{EBV}}}{\sigma_{\mathrm{EBV}}},\;$ ϕ(α) is the standard normal probability density function defined as $\frac{1}{\sqrt{2\pi }}e^{\frac{-\alpha^{2}}{2}},$ and $\Phi \left(\alpha \right)$ is the standard normal cumulative density function defined as $\int_{-\infty }^{\alpha }\phi (t)\mathrm{dt}.$ Furthermore, the probability distribution of $\left(\mathrm{EBV}|\mathrm{EBV}>a\right)$ follows a truncated normal distribution.

Using the law of total expectation and Equations [2] and [4], it can be deduced that the expected mean TBV of a population which has been selected on a minimum EBV (i.e., truncated on EBV) with a given reliability (Rel) is

[6] $$\begin{array}{c} \left(\mathrm{TBV}|\mathrm{EBV}>a\right)=\mu_{\mathrm{TBV}}+\frac{\sqrt{\mathrm{Rel}}\sigma_{\mathrm{TBV}}}{\sigma_{\mathrm{EBV}}}\cdot \frac{\phi \left(\alpha \right)}{1-\Phi \left(\alpha \right)}\sigma_{\mathrm{EBV}}\\ =\mu_{\mathrm{TBV}}+\frac{\sqrt{\mathrm{Rel}}\sigma_{\mathrm{TBV}}\phi \left(\alpha \right)}{1-\Phi \left(\alpha \right)} \end{array}$$

and using the law of total variance and Equations [2], [3], and [5], the expected variance of the distribution of TBV in a population following selection on EBV in that population with a given reliability (Rel) is

[7] $$\begin{array}{c} \left(\mathrm{TBV}|\mathrm{EBV}>a\right)=\;\frac{\mathrm{Rel}\sigma_{\mathrm{TBV}}^{2}}{\sigma_{\mathrm{EBV}}^{2}}\sigma_{\mathrm{EBV}}^{2}\left(1+\frac{\alpha \phi \left(\alpha \right)}{1-\Phi \left(\alpha \right)}-{\left(\frac{\phi \left(\alpha \right)}{1-\Phi \left(\alpha \right)}\right)}^{2}\right)\\ +\left(1-\mathrm{Rel}\right)\sigma_{\mathrm{TBV}}^{2},\\ =\;\mathrm{Rel}\sigma_{\mathrm{TBV}}^{2}\left(1+\frac{\alpha \phi \left(\alpha \right)}{1-\Phi \left(\alpha \right)}-{\left(\frac{\phi \left(\alpha \right)}{1-\Phi \left(\alpha \right)}\right)}^{2}\right)+\left(1-\mathrm{Rel}\right)\sigma_{\mathrm{TBV}}^{2}\\ =\;\sigma_{\mathrm{TBV}}^{2}\left(1+\mathrm{Rel}{\left(\frac{\alpha \phi \left(\alpha \right)}{1-\Phi \left(\alpha \right)}-\left(\frac{\phi \left(\alpha \right)}{1-\Phi \left(\alpha \right)}\right)\right)}^{2}\right). \end{array}$$

The probability distribution of $\left(\mathrm{TBV}|\mathrm{EBV}>a\right)$ is a hidden truncation normal distribution. Equations [6] and [7] can be applied to both the sire and dam populations with the expected mean and variance of the TBV of the resulting (female) progeny being defined as

[8] $$\left(TBV_{Prog}\right)=\frac{\left(\mathrm{TB}V_{\mathrm{sire}}\right)+\;\left(\mathrm{TB}V_{\mathrm{dam}}\right)}{2},$$

[9] $$\left(TBV_{Prog}\right)=\;\frac{1}{4}\left(TBV_{Sire}\right)+\frac{1}{4}\left(TBV_{Dam}\right)+\frac{1}{2}\sigma_{A}^{2},$$

where $\sigma_{A}^{2}$ is the additive genetic variance of the population. The same approach as used to estimate the expected TBV mean and variance of the parental population can also be used to estimate the expected TBV mean and variance of the progeny if selection was applied to just the progeny based on their EBV.

A simulation was undertaken to compare the expected mean and variance of progeny TBV from different scenarios versus those calculated deterministically. A total of 10,000 different dairy herds were simulated, each consisting of 300 candidate dams. The TBV of the dam trait or index were sampled from a normal distribution with a mean of 0 and SD of 1. The genomic EBV of each dam was simulated to have a correlation with the TBV equal to the square root of the reliability of the genomic evaluations. The accuracy of genomic evaluations was assumed the same for all animals. The reliability of genomic evaluations was assumed to be 0.60 for the base scenario but a reliability of 0.80 was also evaluated.

Ten thousand males were also simulated, with the top 20 chosen as candidate sires. As with the dams, the TBV of the males were sampled from a normal distribution with a mean of 0 and a SD of 1.

Each dam was randomly mated to a sire where mating allocations were sampled from a uniform distribution. The TBV of the resulting progeny were calculated as the average TBV of the respective parents plus the Mendelian sampling term sampled from a normal distribution with a mean of 0 and SD of $\sqrt{0.5}.$ The genomic EBV of the progeny was modeled using the same approach as was used to model the genomic EBV of the dams. The within-herd mean and variance in TBV of the progeny from the dams ranked in the top 10%, 20%, …, 90%, and 100% on their genomic EBV was calculated. Similarly, the within-herd mean and variance in TBV of the progeny ranked in the top 10%, 20%, …, 90%, and 100% on their genomic EBV was calculated. These were compared with the expected values derived using the deterministic equations with the same assumptions.

The dams-to-dams selection pathway in many populations (dairy cows; Schaeffer, 2006) was often traditionally the least contributor to genetic gain in a population. The typical low accuracy of selection of the dams in this pathway relative to the other selection pathways (Schaeffer, 2006) was one contributing factor but arguably more so was the relatively poor selection intensity of the dams. Contributing factors in dairy cow populations included (1) the relatively equal sex ratio of male and female calves from conventional semen with only females entering the herd as replacements; (2) the low frequency of multiple births; (3) the relatively poor in-calf rate necessitating more candidate dams to be considered to generate sufficient replacements, especially in seasonal breeding herds; (4) the traditionally high (involuntary) culling rate in dairy herds necessitating a large number of replacements; and (5) the expanding dairy herd size in many territories. The growth of many national dairy herds in developed countries has slowed down, halted, or even reversed. Dairy cow reproductive performance is also improving in many populations (García-Ruiz et al., 2016). These trends all reduce the number of heifers required and, in turn, the number of dams needed to contribute to the next generation. Arguably, however, the 2 main developments that have intensified the requirement for, and interest in, reducing the proportion of the candidate dam herd contributing to the next generation of herd replacements are (1) the greater uptake of Y-sorted sexed dairy semen (Seidel and DeJarnette, 2022), and (2) the growing market for dairy-beef calves (Berry, 2021).

A strong concordance existed in the present study between the deterministic equations and simulations for the (expected) mean and variance of the TBV in the dam and progeny populations for different selection scenarios; considerable variability in the mean progeny TBV per herd did, however, exist (Figure 1), as well as in the variance of the progeny TBV per herd (Figure 2). Given the strong concordance, only the results from the deterministic equations are presented. Changes in expected mean TBV of the female progeny based on the percentage of candidate dams selected is in Figure 1. Also included in Figure 1 is the expected mean TBV of the selected progeny assuming all dams are selected but with a varying percentage of the female progeny selected on their genomic EBV. Irrespective of the population cohort, the greater the selection intensity, the greater the expected mean TBV of the offspring. The expected mean TBV of the female progeny selected from the top 10% of dams based on their genomic EBV (genomic EBV reliability of 60%) was 0.68 SD units higher than the expected mean TBV of the female progeny born from an unselected population of dams; this advantage in TBV of 0.68 SD units increased to 1.26 SD units when all dams were selected, but instead the top 10% of the female progeny ranked on their genomic EBV were selected (Figure 1). The benefit of selection in either population increased as the accuracy of the genomic evaluations of the respective cohort increased (Figure 1); moreover, the difference in mean expected TBV of the progeny between the strategy of selecting dams versus female progeny diverged as the accuracy of the genomic EBV increased (Figure 1). In relative terms, however, the benefit of selecting just the female progeny was consistency 1.86 times that versus selection of the same percentage of just dams, irrespective of either the proportion selected or the accuracy of the genomic evaluations (assuming the accuracy of genomic evaluations was the same for both cohorts). In a supplementary analysis in which the selection intensity of the sires was altered, the relative difference between both selection strategies increased as the genetic variance of the sire population increased (e.g., the proportion of sires selected increased). In fact, if the true genetic variance of the sire and dam population were both 1, then the benefit of selecting just the female progeny would be double that of selecting the same percentage of dams. Although truncation is expected in the sire population, this truncation is often just on a total merit index and the variability in genetic merit for individual traits, especially those not included in the total merit index, could indeed still be large.

**Figure 1:**
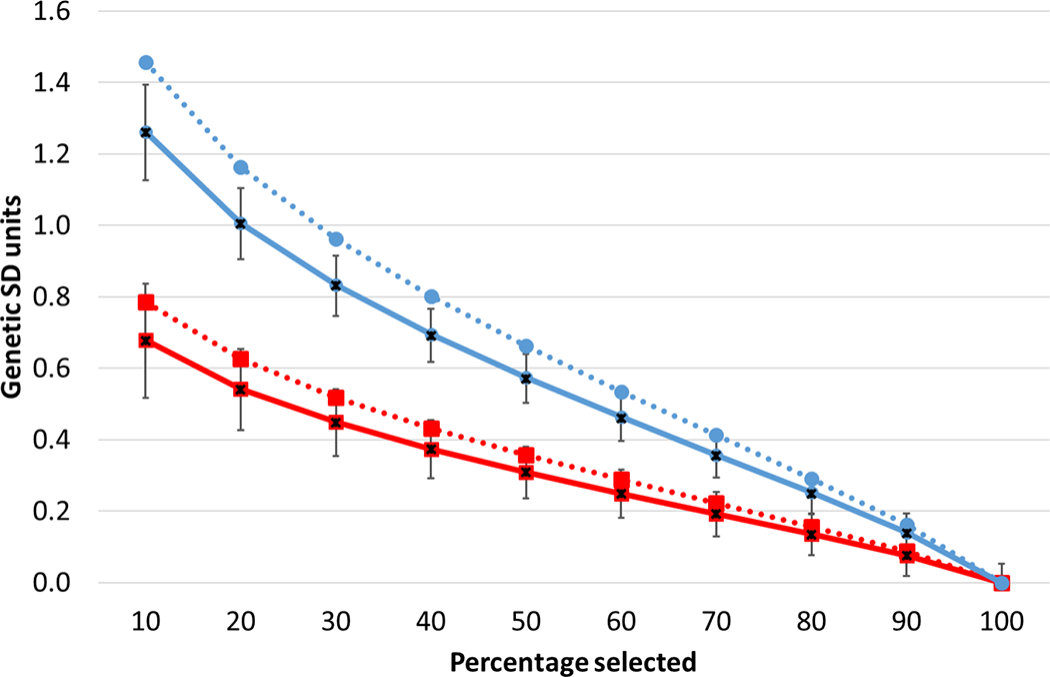
Expected mean true breeding value (in genetic SD units) of the heifer progeny when the top ranking 10%, 20%, …, 90%, 100% of dams (red squares) or heifers (blue circles) are selected on genomic breeding values relative to when no selection is practiced assuming a reliability of genomic evaluations of 60% (continuous lines) or 80% (broken lines). Also included is the mean (x) and SD (1 SD each side of the mean, bars) true progeny breeding value per simulated herd assuming 60% reliability for the selection of the dam or the progeny.

**Figure 2:**
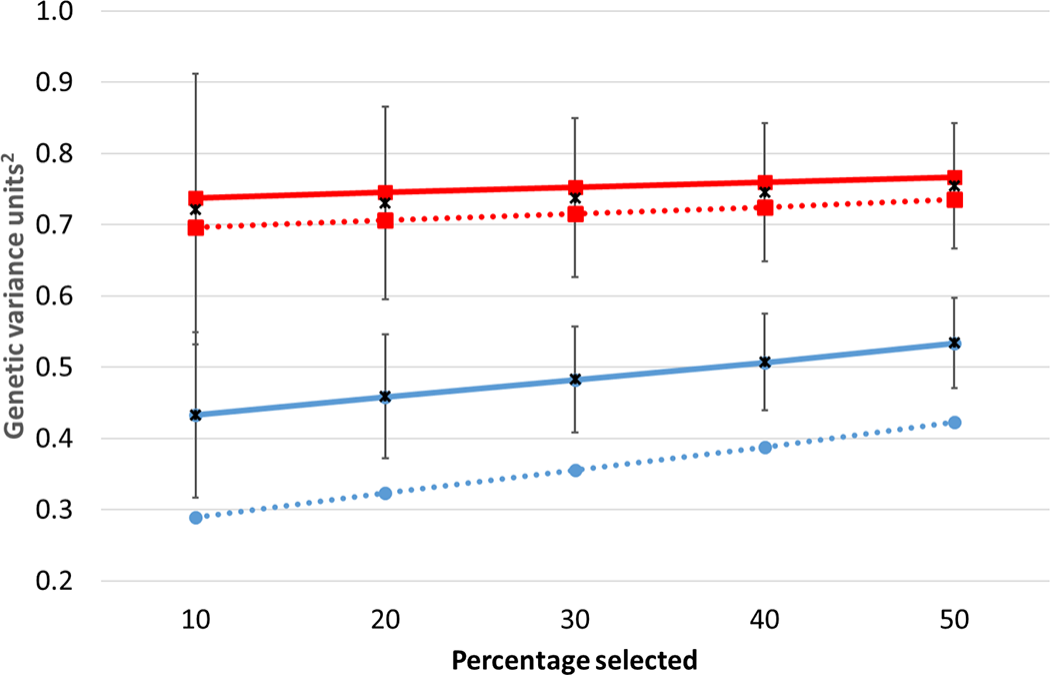
Expected variance of true breeding value (in units^2^) of the heifers when the top ranking 10%, 20%, 30%, 40%, and 50% of dams (red squares) or heifers (blue circles) are selected on genomic breeding values relative to when no selection is practiced assuming a reliability of genomic evaluations of 60% (continuous lines) or 80% (broken lines); 10% of the candidate sire population were chosen as sires. Also included is the mean variance (x) and the SD (1 SD each side of the mean, bars) of the variance of true progeny breeding value per simulated herd assuming 60% reliability for the selection of the dam or the progeny.

The expected variance in TBV of the selected female progeny reduced as selection intensity increased (Figure 2); selection within just the female progeny population resulted in a lower expected genetic variance of the TBV relative to selection only on genomic EBV of the candidate dams. If the genomic reliability of all animals increased, the effect of dam selection on the expected TBV variance in the female progeny was minimal but the impact of female progeny selection did have a noticeable effect on the expected TBV variance of these progeny (Figure 2). It should be noted that these results are relevant only at the herd level and based on just one generation of selection. The practical (i.e., observed) impact of a lower expected (genetic) variability in the female progeny from selection is a function of the heritability of the trait or index under selection. For example, for a trait with a heritability of 10%, then halving the (genetic) variance of the female progeny will equate to only a 5% reduction in the phenotypic variance (after adjusting for systematic environmental effects); halving the genetic variability among the female progeny for a 30% heritable trait is expected to reduce the phenotypic variance by 18%. A reduction in genetic variance can, nonetheless, affect long-term genetic gain if occurring across the entire population, as well as potentially affecting the robustness of the animals to external (novel) perturbations. However, approaches such as selecting candidate parents of the next generation on gametic variance could potentially mitigate this reduction in genetic variance over generations (Bijma et al., 2020).

The effect of combining both dam and female progeny selection on the expected mean TBV of the female progeny is in Table 1; the off-diagonals represent a combination of dam and female progeny selection, whereas the diagonals represent dam selection only. The benefit in expected TBV from a second selection step (i.e., female progeny) was obvious although the benefit of preselecting dams on their genomic EBV reduced as the percentage of dams selected increased. Disregarding the poorer half of candidate dams had little effect on the average predicted TBV of the selected female progeny when only a small proportion of progeny (≤20% of the herd) were chosen for breeding. The greatest expected mean TBV of the selected female progeny was when all dams were selected and only 10% of the resulting heifers were required as replacements; the mean TBV of these female progeny was 1.28 genetic SD units superior to the scenario where the entire dam population was used to generate female progeny and all female progeny were retained. In most cases, however, dairy herds will seek to generate ∼30% of their herd size as replacement heifers; in such a scenario there is a clear benefit from selecting 60% of the candidate dams with the further gains thereafter being relatively small.

**Table 1:** Mean true breeding value in genetic SD units of the heifers resulting from a 2-step selection of the dams, including selecting 100% of dams, followed by selection of the born heifers assuming a 60% reliability of genomic evaluations

Percentage of dams selected	Percentage of heifer replacements required (relative to herd size)									
	10	20	30	40	50	60	70	80	90	100
10	0.68									
20	1.08	0.54								
30	1.18	0.82	0.45							
40	1.23	0.91	0.66	0.37						
50	1.26	0.96	0.75	0.55	0.31					
60	1.27	0.99	0.79	0.62	0.45	0.25				
70	1.28	1.01	0.82	0.66	0.52	0.37	0.19			
80	1.28	1.02	0.84	0.69	0.56	0.43	0.30	0.14		
90	1.27	1.02	0.84	0.70	0.57	0.46	0.34	0.22	0.08	
100	1.26	1.01	0.83	0.69	0.57	0.46	0.36	0.25	0.14	0.00

In all, therefore, the results from the present study, albeit based on just one generation of within-herd selection, support the original hypothesis that the expected mean progeny TBV will be higher if selection is imposed on the female progeny themselves relative to the same selection intensity on their dams. This was simply because of the greater ability of progeny selection to capture the random segregation of alleles during gametogenesis; as observed in this study, the impact is greater when the genetic variance in the sire population is larger (results not shown) since the genetic variance of the sire population contributes quarter the genetic variability in the female progeny and cannot be captured from the genotypes of the dams. Even if the TBV of both parents are known with certainty, the maximum reliability of the parental average EBV of (nongenotyped) newborn progeny is a quarter the sum of the reliability of the parents.

Although several biological assumptions were made in the present study to facilitate the ease of presentation of the calculations, departures from these assumptions can easily be accommodated. The reliability of genomic evaluations is a function of the reference population and prediction algorithms (Hozé et al., 2014) but genomic reliability is already a component in the equations presented. All else being equal and assuming some selection, the greater the reliability of either of the sire, dam or progeny cohort, the greater the expected mean TBV of the progeny. Differences in in-calf rate to sexed semen and its effect on dam selection intensity or the number of serves as well as the proportion of heifers from sexed semen can be accommodated by appropriately changing the truncation point for selecting the candidate dams; this strategy can also be used to accommodate the use of conventional (i.e., not sorted) semen. Calf mortality and infertility of female progeny can similarly be accommodated by altering the truncation point in the distribution of the EBV of the dam. As an example, a static herd with a culling rate of 25% (Bell et al., 2010) could equate to a desire for having possibly 30% of the herd size born as dairy heifers thereby allowing for wastage, including phenotypic selection (e.g., conformation). Assuming sexed semen is used (but is not completely successful in only generating females) but also considering cow infertility or embryo loss, it could be recommended to breed 40% of the candidate dams with dairy-sire semen to generate replacements; the mean expected TBV of the female progeny is 0.37 SD units higher than the if no dam selection was practiced assuming failure to generate a live female calf was random in the dam population. If instead the best 60% of dams (assuming a genomic reliability of 60% for the EBV of the dams) were bred with dairy-sire semen and, assuming infertility and embryo loss is random across dams, and the (genomically) best of the heifers were chosen which represent 30% of the herd size (i.e., the top 60% of the heifers born), then the average TBV of these heifers is expected to be, on average, 0.69 genetic SD units relative to if all dams were used to generate progeny. If sexed semen was not being used, then it could be recommended to breed 80% of the dams with dairy-sire semen to generate replacements with no further (genetic) selection possible; the mean TBV of the female progeny from the top 80% of dams is expected to be 0.14 genetic SD units (relative to no dam selection), considerably less than using sex-sorted semen on the top 40% of dams.

What, however, was not considered in the present study, mainly because of the plethora of possibilities and their respective relative merit or value of each, is the breeding strategies and associated opportunity cost of females surplus to requirements. In many instances, the dams could have been mated to beef bulls to generate beef-on-dairy calves (Berry, 2021), which are generally more marketable and valuable that purebred dairy animals for beef production. If, however, the dairy herd is genetically elite, then it may make more sense to generate surplus female progeny and sell the unselected progeny to (genetically inferior) dairy herds. The equations provided can also be used to calculate the expected mean TBV of the unselected heifers. Depending on the mean genetic merit of the herd relative to the national herd population, these heifers may still excel genetically and could have a high market value. Therefore, the value of these alternative strategies should be considered when making a decision on exactly what proportion of candidate breeding females should be considered as candidate dams.

In conclusion, the rate of genetic gain within a herd will suffer if selection is only imposed on the parents. Basing selection solely on the parents ignores the large genetic variability (i.e., half the genetic variance) in the female progeny that arises from mendelian sampling. Hence, selection of the female progeny based on their EBV will also contribute to a greater mean TBV of the selected progeny. The impact of a reduced genetic variance in the progeny following one round of selection on long-term genetic gain across multiple generations needs further exploration as well as the possible mitigation strategies such as selection of parents based on gametic variance (Bijma et al., 2020). The impact of selection on a combined index of both the expected (additive) genetic merit and expected gametic variance of the parents (Bijma et al., 2020) could also impact the relative difference between selection scenarios. With greater gametic variance, the differential between both strategies could, on average, likely increase. Importantly, the increased genetic merit from the selection strategy proposed in the present study is cumulative and permanent in the descendants as well as often being expressed several times by the progeny themselves. Moreover, though, the relative benefits provided herein are a function of the population parameters assumed and should not be extrapolated to other populations. Many factors, most of which are specific to each herd, should also be considered when deciding the tradeoff between increased genetic merit of the progeny versus the opportunity cost of generating females surplus to requirements.
